# Tetrahydrocurcumin Protects against Cadmium-Induced Hypertension, Raised Arterial Stiffness and Vascular Remodeling in Mice

**DOI:** 10.1371/journal.pone.0114908

**Published:** 2014-12-11

**Authors:** Weerapon Sangartit, Upa Kukongviriyapan, Wanida Donpunha, Poungrat Pakdeechote, Veerapol Kukongviriyapan, Praphassorn Surawattanawan, Stephen E. Greenwald

**Affiliations:** 1 Department of Physiology, Faculty of Medicine, Khon Kaen University, Khon Kaen 40002, Thailand; 2 Department of Pharmacology, Faculty of Medicine, Khon Kaen University, Khon Kaen 40002, Thailand; 3 Research and Development Institute, The Government Pharmaceutical Organization, Rama VI Road, Ratchathewi, Bangkok 10400, Thailand; 4 Pathology Group, Blizard Institute, Barts and The London School of Medicine and Dentistry, Queen Mary University of London, London E1 1BB, United Kingdom; Texas A & M University Health Science Center, United States of America

## Abstract

**Background:**

Cadmium (Cd) is a nonessential heavy metal, causing oxidative damage to various tissues and associated with hypertension. Tetrahydrocurcumin (THU), a major metabolite of curcumin, has been demonstrated to be an antioxidant, anti-diabetic, anti-hypertensive and anti-inflammatory agent. In this study, we investigated the protective effect of THU against Cd-induced hypertension, raised arterial stiffness and vascular remodeling in mice.

**Methods:**

Male ICR mice received CdCl_2_ (100 mg/l) via drinking water for 8 weeks. THU was administered intragastrically at dose of 50 or 100 mg/kg/day concurrently with Cd treatment.

**Results:**

Administration of CdCl_2_ significantly increased arterial blood pressure, blunted vascular responses to vasoactive agents, increased aortic stiffness, and induced hypertrophic aortic wall remodeling by increasing number of smooth muscle cells and collagen deposition, decreasing elastin, and increasing matrix metalloproteinase (MMP)-2 and MMP-9 levels in the aortic medial wall. Supplementation with THU significantly decreased blood pressure, improved vascular responsiveness, and reversed the structural and mechanical alterations of the aortas, including collagen and elastin deposition. The reduction on the adverse response of Cd treatment was associated with upregulated eNOS and downregulated iNOS protein expressions, increased nitrate/nitrite level, alleviated oxidative stress and enhanced antioxidant glutathione. Moreover, THU also reduced the accumulation of Cd in the blood and tissues.

**Conclusions:**

Our results suggest that THU ameliorates cadmium-induced hypertension, vascular dysfunction, and arterial stiffness in mice through enhancing NO bioavailability, attenuating oxidative stress, improving vascular remodeling and decreasing Cd accumulation in other tissues. THU has a beneficial effect in moderating the vascular alterations associated with Cd exposure.

## Introduction

Cd, in its ionic form (Cd^2+^), is a highly toxic metal that is widely distributed in the environment. The risk of human exposure to Cd is steadily increasing through a variety of routes, including industrial contamination, food sources and tobacco smoke [1]. Exposure to Cd can result in a variety of adverse effects in humans and animals. It induces oxidative stress, damaging organs such as kidneys, liver, bone, lungs as well as the endocrine, immune, reproductive and cardiovascular systems [2]–[4]. Interestingly, it has been demonstrated that the vascular system is a critical target of Cd toxicity, leading to an increased risk of cardiovascular disease (CVD) such as hypertension, atherosclerosis and diabetes [5]–[7]. Many epidemiological studies have suggested a positive association between blood Cd^2+^ level and blood pressure in the general population [8]–[11]. Many animal studies have shown that chronic exposure to Cd can lead to elevated blood pressure [12]–[15]. However, the exact biological mechanisms that link Cd exposure and hypertension remain unclear. A marked increase in oxidative stress during Cd exposure affects the vascular cells at a variety of molecular levels, thereby leading to vascular damage and dysfunction [16]–[18]. Furthermore, oxidative stress in the vasculature also reduces the availability of the vasodilator nitric oxide (NO), causes vascular tissue injury and inflammation, as well as promoting lipid, protein and DNA damage [19]. Previous studies suggest that reactive oxygen species (ROS) and reactive nitrogen species (RNS) are important intracellular signaling molecules that regulate vascular function by modulating vascular cell contraction/dilation, growth/apoptosis, migration, and extracellular matrix protein turnover. All of these factors contribute to vascular remodeling and stiffening [20]. Therefore, reducing the presence of ROS, increasing NO bioavailability and augmenting the regression of remodeling and stiffening are important therapeutic strategies against hypertension.

There are several studies describing the use of antioxidant and metal chelating agents for the treatment of metal poisoning. The most important source of antioxidants is provided by nutrition. Apart from their free radical scavenging activities, nutritional antioxidants are known to regulate the expression of number of genes and signal regulatory pathways and thereby to prevent cell death [21]. THU is an antioxidative substance which is derived from curcumin by hydrogenation. Curcumin is a phenolic compound extracted from the rhizome of turmeric (*Curcuma longa* Linn) of the Zingiberaceae family. THU possesses strong antioxidant and free radical scavenging properties [22]–[23] and contains the same phenolic and β-diketo moieties as curcumin. Recently, much attention has been focused on THU, as it appears to exert greater antioxidant activity than curcumin [24]–[27]. THU effectively protects against oxidative stress, endothelial dysfunction, diabetes and hypertension [28]–[30]. It has been reported that THU enhances antioxidant enzyme activities, including superoxide dismutase, catalase and glutathione peroxidase, whereas it decreases thiobarbituric acid reactive substance, hydroperoxide and protein carbonyl formation [24], [26], [28]. Moreover, THU reduced the infarct size in an ischemic–reperfusion model of myocardial infarction [31] and these effects were associated with reduced lipid peroxidation and improved antioxidant status. Therefore, THU may be beneficial for cardiovascular protection, especially against hypertension, by decreasing oxidative stress in the vascular system. Although THU exerts important effects on reduction of blood pressure and arterial stiffening in L-NAME-induced hypertension [30], these valuable effects have not been explored in a model of heavy metal toxicity.

In the present study, we hypothesized that THU could alleviate hypertension, vascular dysfunction and vascular remodeling in mice exposed to Cd. Since ROS can induce endothelial nitric oxide synthase (eNOS) uncoupling and activate MMPs [19], [32], THU as a strong antioxidant may improve NO bioavailability and inhibit MMP activation. Therefore, we have also examined whether THU would attenuate vascular oxidative stress in mice during Cd exposure.

## Materials and Methods

### Chemicals and Drugs

THU (purity>99% w/w by HPLC chromatogram) was synthesized and provided by the Research and Development Institute, The Government Pharmaceutical Organization, Bangkok, Thailand. Phenylephrine (Phe), thiobarbituric acid (TBA), 5,5 dithio-bis-2-nitrobenzoic acid (DTNB), ethylenediamine tetraacetic acid (EDTA), sodium dodecyl sulfate (SDS), glutathione (GSH), butylated hydroxytoluene (BHT), sulfanilamide, dinitrophenylephrinenylhydrazine (DNPH), *N*-1-nepthylethylenediamine dihydrochloride (NED), 1,1,3,3-tetraethoxypropane, bromophenol blue, 2-mercaptoetanol, lucigenin and guanidine were purchased from Sigma-Aldrich Pte. Ltd. (Singapore). Nitrate reductase, nicotinamide adenine dinucleotide phosphate (NADPH) and glucose-6-phosphate dehydrogenase were obtained from Roche Applied Sciences (Mannheim, Germany). Acetylcholine chloride (ACh), sodium nitroprusside (SNP), trichloroacetic acid (TCA), metaphosphoric acid (MPA), 1-methyl-2 vinyl-pyridinum trifate (M2VP), lucigenin, Tween and skimmed milk were obtained from Fluka Chemika Co. Ltd (Buch, Switzerland). Mouse monoclonal anti-eNOS was obtained from BD Biosciences (CA, USA). Anti-mouse IgG antibody, a rabbit polyclonal anti-inducible nitric oxide symthase (iNOS) antibody and anti-rabbit IgG antibody were purchased from Santa Cruz Biotechnology (Santa Cruz, CA, USA). All other chemicals used were of analytical grade quality.

### Animals and Experimental Protocols

Adult male ICR mice weighing 25–30 g were obtained from the National Laboratory Animal Center, Mahidol University, Salaya (Nakornpathom, Thailand). The animals were housed at The Northeast Laboratory Animal Center (Khon Kaen University, Thailand) and maintained on a 12-h dark/light cycle at room temperature (25±2°C) with free access to standard rat chow (Chareon Pokapan Co. Ltd., Thailand). This study was carried out in strict accordance with the recommendations in the Guide for the Care and Use of Laboratory Animals of the National Institutes of Health. The protocol was approved by the Committee on the Ethics of Animal Experiments of the Khon Kaen University (Permit Number: AEKKU42/2554). All surgery was performed under the standard anesthetic drugs, and all efforts were made to minimize suffering.

After 1 week of acclimatization, mice were randomly divided into six experimental groups (N = 8–10/group). Group I, normal controls, received deionized water (DI) as drinking water and were treated with polyethylene glycol (PG), the THU vehicle, administered intragastrically at 1.5 ml/kg/day. Groups II and III, THU-controls, received DI and were treated with THU at doses of 50 and 100 mg/kg/day respectively, administered intragastrically 5 days/week for 8 weeks. Groups IV, V, and VI received DI containing CdCl_2_ (100 mg/l) in their drinking water and administered THU at doses of 50 or 100 mg/kg/day (dissolved in PG), again for 5 days/week for 8 weeks. THU was kindly provided by the Government Pharmaceutical Organization (Bangkok, Thailand). The doses of Cd and THU were chosen on the basis of previous studies [30], [33].

### Measurement of Blood pressure, Heart rate and Assessment of Vascular Reactivity

At the end of the treatment period, the animals were anesthetized with an intraperitoneal injection of ketamine (100 mg/kg) and xylazine (2.5 mg/kg). Blood pressure and vascular reactivity were determined as previously described [14]. A tracheotomy was performed for spontaneous breathing. The animal's body temperature was kept constant at 37±2°C by a heating pad. The right carotid artery was exposed and cannulated with polyethylene tubing connected to a pressure transducer for monitoring arterial blood pressure and heart rate using the Acqknowledge data acquisition system (Biopac System Inc., CA, USA). The left jugular vein was exposed and cannulated with polyethylene tubing for infusion of vasoactive agents. After obtaining baseline measurements, ACh (10 nmol/kg), an endothelium-dependent vasodilator, SNP (10 nmol/kg), an endothelium-independent vasodilator, and Phe (0.03 µmol/kg), an alpha sympathomimetic agent, were randomly infused intravenously, while blood pressure was continuously monitored. Following the drug infusion, blood pressure was allowed to return to the baseline level and stabilize for at least 5 minutes. Changes in blood pressure were expressed as percentages of control values obtained immediately before administration of the test substance (baseline). At the end of the experiment, the animals were sacrificed by anesthetic overdose, blood samples were collected from the abdominal aorta for assays of the antioxidant GSH and oxidative stress makers (see below). Thereafter, the aorta was rapidly excised and the amount of superoxide (O_2_^•-^) production in each specimen was measured. In a separate set of experiments, blood and tissues of each animal were collected for assessments Cd content, aortic elasticity and vascular remodeling.

### Assays of Nitrate/nitrite, Oxidative Stress Markers and the Antioxidant Glutathione

Accumulation of nitrate and nitrite, the oxidative products of NO, was measured in urine samples using a previously described method [14]. The production of O_2_^•-^ in mouse aorta was determined by the lucigenin-enhanced chemiluminescence method as previously described [14]. The malondialdehyde (MDA), a lipid peroxidation marker, was assessed in the plasma, heart, kidney and liver using the TBA assay [33]. The protein carbonyl, a protein oxidation marker, in the plasma and tissues, was assessed by the determination of carbonyl groups based on the reaction with DNPH as previously described [33]. MDA and protein carbonyls concentrations in the tissues were normalized against the tissue protein concentration, which was determined by the Bradford dye binding method [34]. GSH in the blood was determined spectrophotometrically, again following a previously described method [35].

### Western Blot Analysis

NO, normally synthesized by eNOS plays a role in the regulation of vascular function. However, under conditions of stress, such as inflammation, several oxidative stressors can induce the expression of iNOS, which thereby induces NO production [36]. Therefore, we determined both eNOS and iNOS protein expression in the aortas of all studied animals, by Western blotting as previously described [37]. The expressions of eNOS and iNOS were normalized to β-actin expression from the same sample. The data were expressed as a percentage of normal controls.

### Assessment of Aortic Elasticity

In a separate set of experiments, after the animals were killed by overdose of the anesthetic drug, the quasi static aortic elasticity (n = 6-8/group) was assessed. In brief, the thoracic aorta was cannulated in situ with polyethylene catheters, one distal to the arch and the other just below the diaphragm. The aorta was perfused with barium sulfate through the thoracic cannula and inflated at varying pressures ranging from 0-200 mmHg at 20 mmHg intervals. Each pressure was allowed to stabilize for 30 seconds and an image of the aorta filled with the barium sulphate, which helped localize small leaks and improved the contrast of the aorta against the surrounding tissue, was taken with a digital camera fitted with macro lens (Canon EOS 40D, Canon Marketing (Thailand) Co., Ltd., Bangkok, Thailand) The external diameter of the aorta was derived from these images with the aid of Image-Pro Plus software (Media Cybernetics, Silver Spring, MD, USA). Firstly, the area of each segment of the aorta measured at each perfusion pressure was determined by tracing the edges of the vessel with the computer mouse and determining the ferret length of the outlined area. Knowing the length of the outlined image, D_e_ of the aorta at various pressures was calculated by dividing the area by the length. For each aortic segment the inflation was performed twice and the two diameter readings at each pressure were averaged.

After completing the inflation experiment and flushing out the barium sulfate, the in-situ length of the thoracic aorta was measured, after which it was excised from the animal and weighed. Knowing the volume of the vessel from its weight and its length in situ, the cross-sectional area (CSA) can be calculated from; CSA  =  Mass/Length x ρ, where density of the tissue (ρ) is 1.06 mg/mm^3^. The wall thickness (h) of the vessel was calculated as the difference between the by external radius (R_e_) and internal radius (R_i_). R_e_ was taken as D_e_/2. and Ri was derived from the expression: R_i_ =  [(D_e_/2)^2^ – (CSA/π)]½. Midwall radius (R_m_) was calculated as; R_m_ = R_e_ -(h/2). The relative wall thickness was used to normalize h with R_m_ at each pressure. Langewouter's equation [38] was used to fit the data points to obtain the pressure-diameter relationship in analytical form from which the gradient at any point could be derived. The functional stiffness (Peterson's elastic modulus) (Ep) and material stiffness (the circumferential incremental elastic modulus) (E_inc_) were calculated from the relative change in blood vessel radius in response to a known change in pressure following the equations; E_p_ =  (ΔPR_m_^2^)/(ΔR_m_h) and E_inc_  = 0.75 (ΔPR_m_^2^)/(ΔR_m_h), where ΔP is the pressure increment and ΔR is a change in radius due to ΔP. Relative radius (ΔR_m_) or circumferential extension ratio was used to normalize R_m_ with R_m_ at the pressure of 0 mmHg.

### Morphometric Analysis and Composition of the Vascular Wall

The middle portion of the descending thoracic aortas were cleaned, fixed in 4%phosphate-buffered paraformaldehyde, pH 7.4, and embedded in paraffin blocks. Slices, 4 µm thick were stained with hematoxylin and eosin (H&E), Picrosirius red, and Miller's elastic stain to determine the number of smooth muscle cells (SMCs), the area fractions of collagen and elastin in the aortic media, respectively [39]. The number of SMCs was obtained by counting their nuclei in the sections stained with H&E. The area fraction of collagen or elastin in the aortic media was assessed by counting threshold pixels stained with Picrosirius red or Miller's elastic staining and dividing by the total number of medial pixels. The stained sections were examined with light microscopy and the image was captured at ×400. The composition of the aortic wall was evaluated using KS400 image analysis system (Carl Zeiss Microscopy, Imaging Associates Ltd., Bicester, UK).

### Immunohistochemistry to Detect Vascular MMP-2 and MMP-9

In order to assess MMP-2 and MMP-9 localization in the thoracic aorta, the de-waxed aortic sections were analyzed using specific antibodies (ab37150; Abcam and ab19016; Millipore, respectively), and an immunohistochemical technique using the R.T.U. Vectastain ABC kit (Vector Laboratories, Inc., CA, USA) as previously described [40]. The experimental conditions were optimized to generate a strong and specific signal for the antigen of interest. The measurements were performed in a single batch and the specimens were stained together with a positive control at the same time. The stained sections was examined with light microscopy and the images were captured at ×400. The amount of MMP2 or MMP-9 in the aortic wall was measured, as were collagen and elastin, by counting the thresholded pixels stained with antibodies to MMP-2 or MMP-9 using the Image-Pro Plus Program (Media Cybernetics, MD, USA). All measurements were made by one observer and preliminary observations of intra-observer repeatability showed a coefficient of variation of less than 5% for all estimations of thresholded area.

### Cd Assay

Cd content in the blood and tissues, including the aorta, heart, liver and kidney was determined as previously described [35] using inductively coupled plasma mass spectrometry (ICP-MS) (Agilent 7500 ICP-MS model, Santa Clara, CA, USA) according to the manufacturer's recommendation. The Cd contents were expressed in µg/l and µg/g of tissue wet weight.

### Statistical Analysis

Results are expressed as mean ± SEM., and n refers to the number of animals used. Statistical evaluation was performed by one-way analysis of variance (ANOVA) followed by Newman–Keuls post-hoc test to show specific group differences. Statistical significance was determined at a level of *P*<0.05.

## Results

### THU Attenuates Cd-Induced Hypertension and Vascular Dysfunction

Arterial blood pressure (BP) and heart rate (HR) of all experimental groups are shown in Table 1. Systolic blood pressure, diastolic blood pressure and mean arterial pressure were increased in mice exposed to Cd (*P*<0.05). Supplementation with THU significantly decreased blood pressure when compared with mice receiving Cd alone in a dose dependent manner (*P*<0.05). There was no change in HR among all groups (Table 1).

**Table 1:** Effect of THU on arterial blood pressure and heart rate in all experimental groups.

Variables	Untreated control	Control + THU (mg/kg)		Cd control	Cd + THU (mg/kg)	
		50	100		50	100
Systolic pressure (mmHg)	117±3	122±2	123±2	160±2 ^*^	139±2 ^*†^	129± 2^*†^
Diastolic pressure (mmHg)	81±3	76±2	82±4	119±2 ^*^	101±2 ^*†^	80±2^†^
MAP (mmHg)	96±3	97±1	101±2	142±3 ^*^	119±2^ *†^	100±1^†^
Heart rate (beats/min)	336±11	339±7	336±13	336±8	341±14	333±9

Data are expressed as mean ± SEM., n =  8–10/group. MAP, mean arterial pressure. *P<0.05 compared with normal control group, ^†^ *P*<0.05 compared with Cd control group.

**Figure 1:**
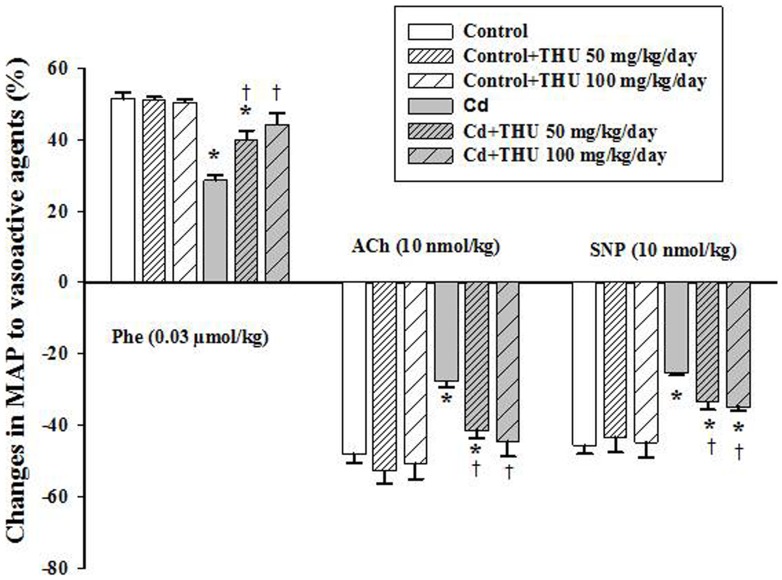
Effect of THU on alteration of blood pressure in response to vasoactive agents in all experimental groups. Change in mean arterial pressure (MAP) was calculated as a percentage of baseline MAP after challenge with each vasoactive agent. Results are expressed as mean ± SEM., n = 8–10/group. ^*^ *P*<0.05 compared with untreated control group; ^†^ *P*<0.05 compared with Cd control group.

Vascular hyporesponsiveness to Phe, ACh and SNP was found in mice exposed to Cd (*P*<0.05; Fig. 1), indicating that vasodilating and vasoconstricting properties are impaired during Cd exposure. Interestingly, impairment of vascular responsiveness was largely prevented by THU as shown in Fig. 1. THU, especially at 100 mg/kg significantly increased vascular responsiveness to Phe (44% vs. 28%), ACh (44% vs. 27%), and SNP (34% vs. 25%) when compared to the behaviour of mice receiving Cd alone (*P*<0.05; Fig. 1).

### THU Prevents Cd-Induced Alterations in NO

**Figure 2:**
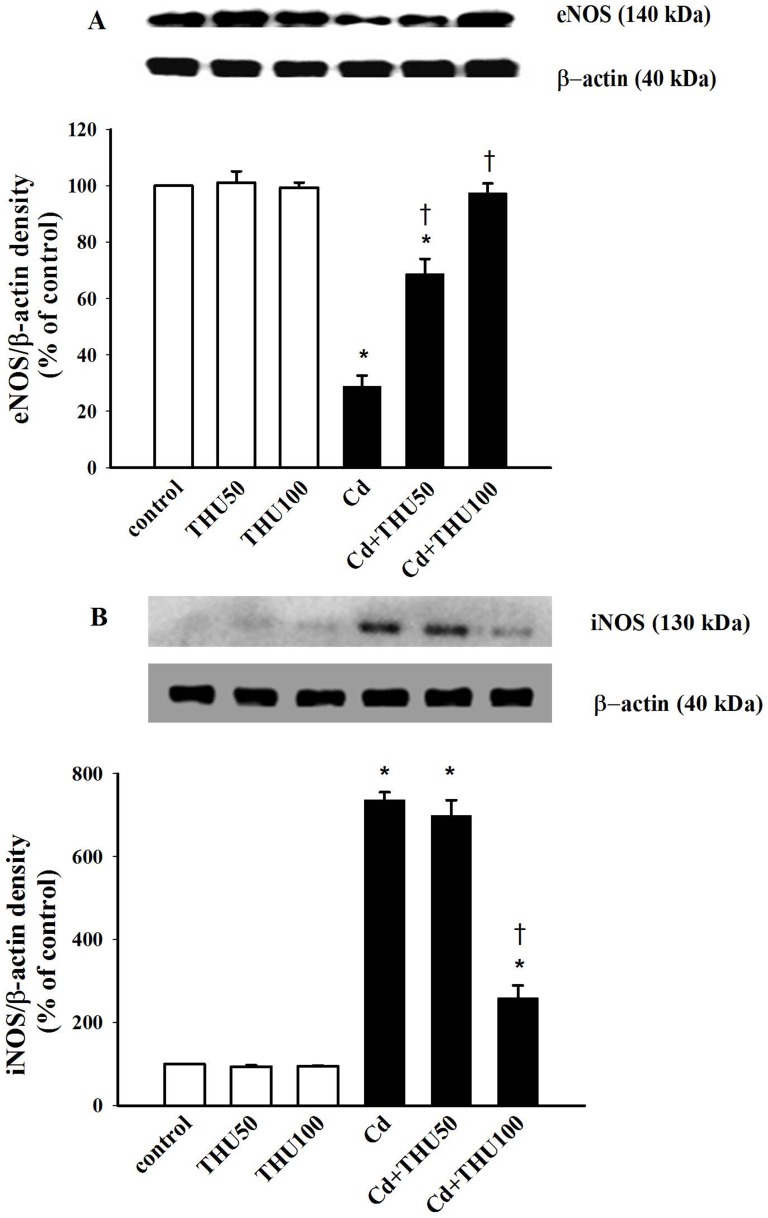
Effect of THU on eNOS (A) and iNOS (B) protein expressions in the aortas of mice in all experimental groups. Plots show the densitometric intensities of eNOS and iNOS protein expressions for each condition, normalized against β-actin expression and presented as percentage of untreated control values. Results are expressed as mean ± SEM., n = 5–6/group. ^*^ *P*<0.001 compared with normal control group; ^†^ *P*<0.001 compared with Cd control group.

Cd profoundly suppressed eNOS but enhanced iNOS expression in mice aortas (*P*<0.001; Fig. 2A, B), revealing the alteration of the eNOS/iNOS pathway in Cd exposure. Moreover, a marked increase in urinary nitrate/nitrite was found in Cd exposed mice (*P*<0.001; Table 2), and this was associated with the increase in iNOS protein. Interestingly, it is apparent that THU upregulated eNOS and downregulated iNOS protein expressions in the aortic tissue and decreased urinary nitrate/nitrite level in Cd exposed mice. THU at both doses did not modify eNOS and iNOS expressions in the aortas of non Cd treated controls.

**Table 2:** Effect of THU on oxidative stress and redox status in all experimental groups.

Parameters	Untreated control	Control + THU (mg/kg)		Cd control	Cd + THU (mg/kg)	
		50	100		50	100
**Aortic superoxide anion** (Counts/mg dry wt./min)	126±7	123±5	121± 8	1060±86^*^	436± 28^*†^	233±18^*†^
**Blood GSH** (µM)	810±70	775±85	806±82	285±24^*^	528±32^*†^	752±80^†^
**Urinary nitrate/nitrite** (nmol/mg creatinine)	881±87	819±115	864±153	1866±218^*^	1463±246^*†^	1014±111^*†^
**Malondialdehyde**						
Plasma(µM)	14±0.4	13±0.5	12±0.7	39±2^*^	38± 1.3^*^	15±1^†^
Heart (µmol/mg protein)	7± 0.7	6±0.6	6.± 0.1	10±0.1^*^	9±0.5^*^	6±0.6^†^
Liver (µmol/mg protein)	3±0.8	3±0.9	3±0.3	7±0.5^*^	5±0.7^*†^	3±0.3^†^
Kidney (µmol/mg protein)	5±0.7	6±1.1	5.±0.6	9±0.3^*^	7±0.2^*†^	5± 1^†^
**Protein carbonyl** (nmol/mg protein)						
Plasma	1.4±0.1	1.5±0.3	1.5±0.7	4±0.4^*^	2.4±0.3^*†^	1.2±0.4^†^
Heart	14± 3	13±5	14±5	25±3^*^	18±01^*†^	16± 0.2^†^
Liver	22±5	25±4	21±3	31±3^*^	25±3^*†^	21±4^†^
Kidney	12±3	13±1	12±3	18±0.8^*^	13±2^†^	13±3^†^

GSH, Glutathione. Data are expressed as mean ± SEM., n = /group. n =  8–10/group, *P<0.05 compared with normal control group, ^†^ *P*<0.05 compared with Cd control group.

### THU Ameliorates Cd-Induced Arterial Stiffness

**Figure 3:**
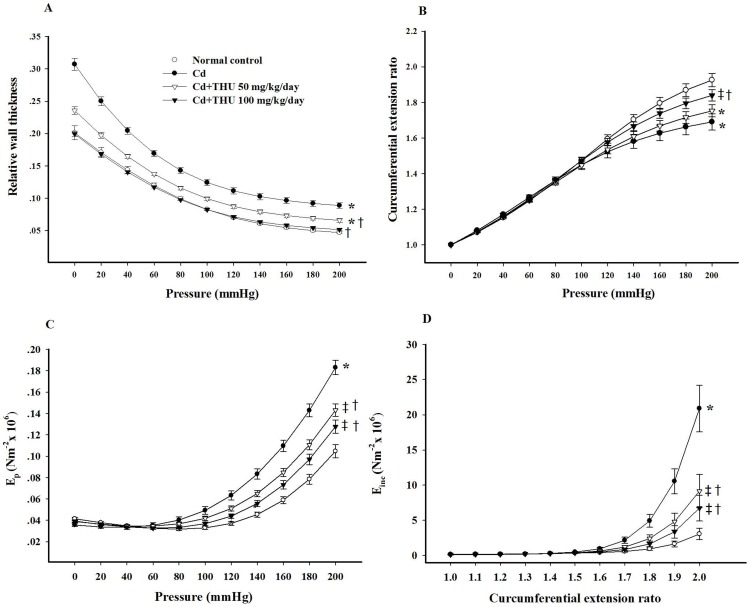
Effect of THU on the relative wall thickness (A), circumferential extension ratio (B), E_p_ (C), and E_inc_ (D) of mice thoracic aortas in all experimental groups. Results are expressed as mean ± SEM., n = 6–8/group. In animals not treated with Cd, THU has no effect on aortic elasticity of normal control mice (data not shown). (A)^*^ *P*<0.05 compared with normal control group at various pressures. ^†^ *P*<0.05 compared with Cd control group at various pressures. (B)^*^ *P*<0.05 compared with normal control group at pressures ranging from 160–200 mmHg; ^‡^ *P*<0.05 compared with normal control group at pressures ranging from 180–200 mmHg); ^†^ *P*<0.05 compared with Cd control group at pressures ranging from 160–200 mmHg. (C)^*^ *P*<0.001 compared with normal control group at pressures ranging from 100–200 mmHg; ^‡^ *P*<0.5 compared with normal control group at pressures ranging from 120–200 mmHg; ^†^ *P*<0.05 compared with Cd control group at pressures ranging from 120–200 mmHg. (D)^*^ *P*<0.05 compared with all group (from 1.7); ^‡^ *P*<0.05 compared with normal control (from 1.9); ^†^ *P*<0.05 compared with Cd control group (from 1.7).

The elastic properties of animals administered vehicle only and those given THU dissolved in the vehicle did not differ. Therefore, only the data of the vehicle-alone controls and the Cd- treated groups with or without THU co-administration are displayed in Fig. 3. The aortic relative wall thickness of mice exposed to Cd was significantly greater than normal controls over the entire range of distending pressures (*P*<0.001; Fig. 3A), indicating that Cd induced vascular structural changes. The circumferential extension ratio of the thoracic aorta of Cd-treated mice was diminished at pressures ranging from 160–200 mmHg although not at physiological pressures (*P*<0.05; Fig. 3B), suggesting a reduction in aortic compliance after Cd exposure. Supplementation with THU ameliorated the vascular structural changes and increased aortic compliance towards control values, as shown in Fig. 3A, B.

Fig. 3C and D show the relationships of E_p_ with pressure and E_inc_ with circumferential extension ratio, respectively. Cd significantly shifted the curves to the left which implies that E_p_ (Fig. 3C) and E_inc_ (Fig. 3D) were increased after Cd exposure indicating that Cd causes increases in both functional and structural aortic stiffness. THU at both doses significantly reduced the aortic stiffness in Cd-treated mice (*P*<0.001; Fig. 3C, D), although at physiological pressures the reduction was not sufficient to reach control values.

### THU Improves Cd-Induced Vascular Remodeling

**Figure 4:**
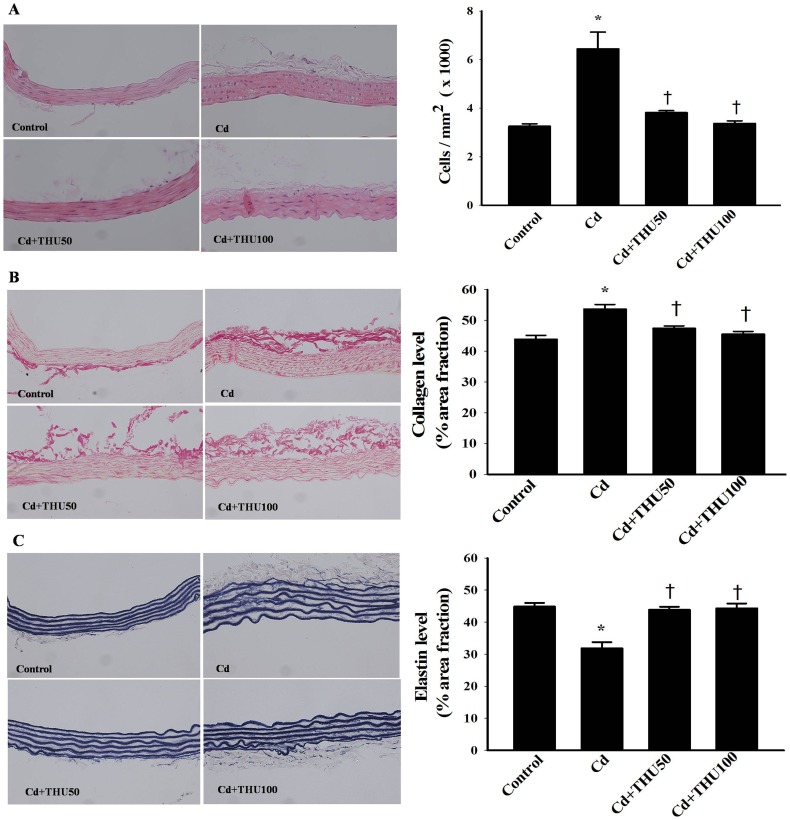
Effect of THU on smooth muscle cell numbers and percent of area fraction of collagen and elastin content in the aortic media of mice with or without Cd exposure. Panels A, B and C are representative photographs of aortic samples (×250) stained with H&E, picrosirius red and Miller's elastic stain, respectively. Results are expressed as mean ± SEM., n = 6–8/group. ^*^ *P*<0.05 compared with normal control group; ^†^ *P*<0.05 compared with Cd control group.

When compared to the untreated controls, Cd-induced hypertension was associated with arterial wall hypertrophy, a significant increase in the number of VSMCs and increased collagen content in the aortic wall (*P*<0.05; Fig. 4A, B).

In addition, aortic elastin content was decreased in Cd-treated mice (*P*<0.05; Fig. 4C), a result in keeping with the Cd-associated increase in arterial stiffness. THU treatment, especially at high dose, inhibited the vascular structural alterations induced by Cd (*P*<0.05; Fig. 4).

**Figure 5:**
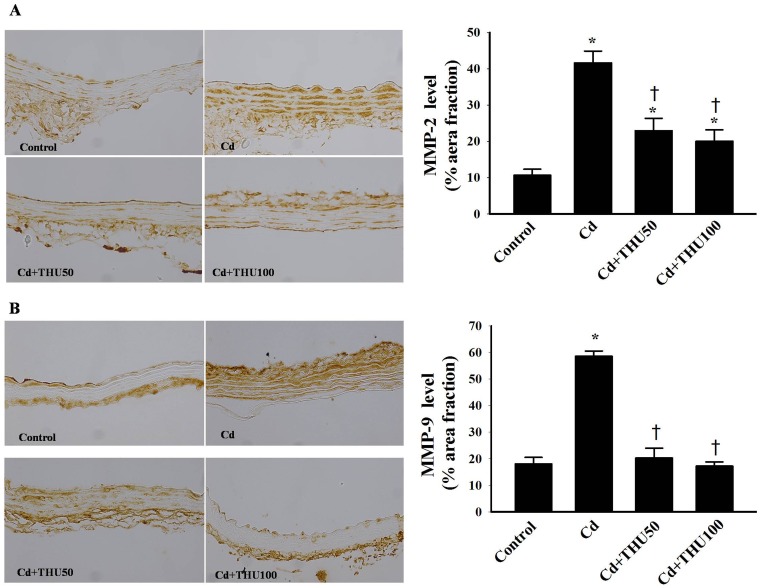
Effect of THU on MMP-2 (A) and MMP-9 (B) localization assessed by immunohistochemistry in the aortas of all experimental groups. Panels A and B are representative photographs of MMP-2 and MMP-9 expressions respectively (400×). Results are expressed as mean ± SEM., n = 6–8/group. ^*^ *P*<0.001 compared with normal control group; ^†^ *P*<0.001 compared with Cd control group.

Representative immunohistochemistry photomicrographs showing MMP-2 and MMP-9 levels in the mouse aortas are shown in Fig. 5. Significantly higher MMP-2 (41.6% vs. 10.6%; Fig.5A) and MMP-9 (58.8% vs. 18%; Fig. 5B) levels were found in the aortas of mice exposed to Cd compared with unexposed controls (*P*<0.001). Interestingly, there was a marked decrease in MMP-2 and MMP-9 levels (approaching normal values) in Cd-exposed mice treated with THU (*P*<0.001; Fig. 5A, B). In addition, there was no significant difference in the aortic wall composition and MMP-2 and MMP-9 levels in normal control mice after supplementation with THU (data not shown).

### THU Alleviates Cd-Induced Oxidative Stress

Table 2 shows the levels of O_2_^•-^, oxidative stress markers and antioxidant GSH in all experimental groups. Results showed that administration of THU did not change the levels of oxidant and antioxidant in control mice when compared with those receiving the vehicle alone. Cd exposure for 8 weeks induced a marked production of O_2_^•-^ in the thoracic aorta and reduced blood GSH when compared to those of normal controls (*P*<0.05; Table 2). THU at both doses significantly reduced the rate of O_2_^•-^ production when compared to mice treated with Cd alone (*P*<0.05; Table 2). Similarly, treatment with THU, especially at high dose, showed an impressive recovery of blood GSH compared to Cd-treated controls (*P*<0.05; Table 2). Further confirming increased oxidative stress associated with Cd-induced hypertension, we found that mice treated with Cd showed higher MDA levels in plasma and tissues, including heart, liver and kidney than the Cd-treated controls (*P*<0.05; Table 2). In addition, increased protein carbonyl levels in plasma and tissues were found in mice treated with Cd (*P*<0.05; Table 2). THU dose-dependently reduced both MDA and protein carbonyl levels (*P*<0.05; Table 2). It appears that the antioxidant activity of THU is well correlated with a decrease in blood pressure and improvement in the vascular responsiveness of Cd-treated mice.

### THU Reduces Cd Accumulations in Blood and Tissues

**Figure 6:**
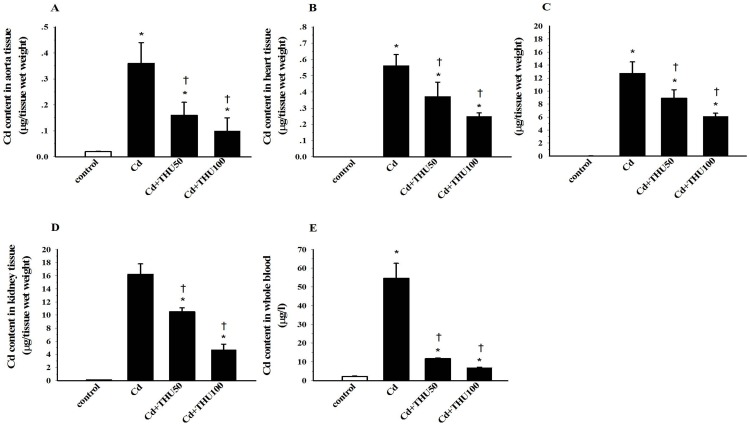
Effect of THU on Cd accumulation in the blood and tissues of mice exposed to Cd. Results are expressed as mean ± SEM., n = 6-8/group. ^*^ *P*<0.05 compared with normal control group, ^†^ *P*<0.05 compared with Cd control group.

A marked increase in Cd content was found in blood and tissues, including heart, aorta, liver and kidney of mice after an 8-week exposure (Fig. 6A, B, C). Supplementation with THU in a dose-dependent manner significantly decreased Cd accumulations in blood and the tissues as shown in Fig. 6.

To test that the mode of action of THU is not merely due to the interference of gastrointestinal absorption, we have performed some supplementary experiments by giving Cd and THU to mice directly via intravenous injection. Data obtained from mice acutely exposed to Cd by intravenous injection showed a significant increase in arterial blood pressure, and THU reduced systolic blood pressure with a slight decrease in mean arterial pressure (S1 Figure). THU also reduced oxidative stress by decreasing vascular superoxide production and the levels of MDA in plasma and tissues. Interestingly, the amount of Cd in the blood of mice receiving Cd directly via intravenous injection was also elevated and declined after THU treatment (S1 Figure). Data from both acute and sub-chronic exposures to Cd suggest a potential chelating effect of THU.

## Discussion

The major findings of this study are that Cd exposure induces hypertension, vascular dysfunction and vascular stiffening by remodeling of the vascular scleroproteins and increased oxidative stress. The consequences of Cd exposure include blunted vascular responses to vasoactive substances, reduced arterial compliance and decreased endothelial NO activity due to a reduction in NO bioavailability. We have found that administration with THU led to a significant reduction in the detrimental effect of Cd-induced hypertension and vascular injury in mice. The plausible mechanisms might be related to the ability of THU to restore the NO bioavailability in the vascular system, suppress MMP-induced vascular remodeling, reduce oxidant formation and maintain antioxidant GSH. In this study, increased arterial blood pressure in mice with Cd exposure, corroborates previous reports that Cd is a risk factor for hypertension [2], [7], [10], [33], [35], [41]. Cd perturbs vascular health and interferes with vascular function [7], [17]. Previous studies suggest that Cd increases oxidative stress and attacks endothelium and VSMCs in a variety of ways, thereby leading to vascular damage and abnormal function [16]–[17], [42]–[43]. This study has confirmed that Cd exposure induces hypertension which may both be the cause and the consequence of functional and structural alterations in the aorta. We found that Cd induced vascular dysfunction by attenuating the response of VSMC to vasoactive agents. The blunted response of Phe-induced contraction reported here may be explained by Cd provoking enhanced Ca^2+^- channel antagonism and thus, resulting in the reduction of Phe contraction [44]. The suppression of the vascular response to ACh after Cd exposure can be explained by decreasing endothelium-dependent vaso-relaxation which is due to down-regulation of eNOS expression, as found in this study, as well as an NO-dependent pathway dysfunction in the vascular endothelium [15]. Moreover, the attenuation of vascular reactivity to SNP observed here might be due to a long-term damage of VSMCs caused by Cd intoxication.

Apart from BP elevation, we also found that the aortic wall of Cd exposed mice becomes thicker and stiffer than normal, and that these alterations are associated with changes in vessel composition. Therefore, changes in mechanical forces during Cd exposure might lead to adaptive restructuring of the vessel wall.

Reorganization of the extracellular matrix through protein synthesis and degradation is a key characteristic of hypertensive vascular remodeling [45]–[46]. Recent studies have shown evidence of MMP activation via NADPH oxidase-derived ROS leading to increased mechanical stretch [47]–[48]. In this study, enhanced expression of arterial MMP-2 and MMP-9 has been observed in mice on exposure to Cd, and these findings are associated with increased BP and modifications of vascular wall composition. Our results are in agreement with previous studies showing that Cd increased MMP-9 and MMP-2 levels which induced inflammation and proliferation [49]–[50]. Therefore, we suggest that increased MMP activation and reduced arterial compliance may very well contribute to hypertensive remodeling during Cd exposure.

The role of nutritional compounds in the prevention and treatment of hypertension has attracted considerable interest over the last decade. Among these, THU, a strong antioxidant, has been shown to exert antihypertensive activity and reduce aortic stiffening [30].

It is well established that NO produced from endothelial cells possesses diverse bioactivity, including smooth muscle relaxation and vasodilation, antioxidant properties, platelet disaggregation, anti-inflammatory and anti-thrombogenic activity, and the inhibition of cell growth, proliferation and migration [51]. Reduction in NO bioavailability may be caused by decreased eNOS expression [52], a lack of substrate or cofactors for eNOS [53], alteration of cellular signaling such that eNOS is not appropriately activated and finally, accelerated NO degradation by ROS. In general, a major source of NO in the vascular system is from eNOS. The present results show a significant decrease of aortic eNOS expression in the aortas of Cd exposed mice. This is in agreement with previous studies that found decreased eNOS levels in the blood vessels of Cd-induced hypertensive rats [15]. Reduced eNOS expression in this study may be due to the downregulation of eNOS gene expression. Cd-induced vascular dysfunction would, in addition to damaging the integrity of the vascular endothelium, promote vascular inflammation [5]. A variety of proinflammatory mediators such as TNF-α may activate iNOS and generate high concentrations of NO-mediated disorders [54]. Overproduction of NO via the iNOS pathway was found in this study. We suggest that iNOS-generated NO suppresses the expression of eNOS, suppresses guanylate cyclase activity and thereby induces a state of endothelial and smooth muscle dysfunction [55]. Altogether, our results show that THU can restore vascular function and structure associated with the eNOS/iNOS regulatory pathway in Cd-induced hypertensive mice.

Cd causes oxidative stress by inducing the generation of ROS and RNS, reducing the antioxidant defense systems of cells by depleting GSH, inhibiting SH-dependent enzymes, interfering with some essential metals needed for antioxidant enzyme activities, and/or increasing susceptibility of cells to oxidative attack by altering the membrane integrity and fatty acid composition [56]–[57]. Cd exposure is associated with increased production of ROS, especially O_2_^•-^ [3]. Moreover, increased O_2_^•-^ production can induce iNOS expression through NF-κB activity which in turn may augment NO production [58]. As shown in our results, Cd exposure causes an increase in O_2_^•-^ production and iNOS expression in the thoracic aorta. Induction of iNOS produces excessive amounts of NO, which reacts with O_2_^•-^ to form the even more potent oxidant peroxynitrite (ONOO^-^). Both O_2_^•-^ and ONOO^-^ have been implicated in tissue injury and dysfunction of organs, including blood vessels, heart, liver, and kidney. Treatment with THU markedly decreased O_2_^•-^ production in the aorta and decreased the ratio of urinary nitrate/nitrite in a dose-dependent manner. Decreased iNOS expression was related to a decline in O_2_^•-^ and nitrate/nitrite levels, thereby, reducing ONOO^-^ formation and causing increased NO bioavailability.

It is known that heavy metals, such as Cd, produce many of their adverse effects by the formation of free radicals, resulting in DNA damage, lipid peroxidation, and depletion of protein sulfhydryls (e.g. GSH) [3], [59]. Our results have shown significant suppression of oxidant formation by THU as indicated by inhibition of the high levels of O_2_^•-^, MDA and protein carbonyl, whereas endogenous antioxidant GSH was increased by THU administration in Cd exposed mice. These results suggest that vascular protection in the THU-treated group is probably due to suppression of oxidative stress. Histological examination further confirmed the protective effect of THU on Cd-induced oxidative stress and vascular toxicity. Previous studies have shown the antihyperlipidemic and cardiovascular protective effects of THU [24], [28], [31]. Pari and Amali have reported that THU confers significant protection against chloroquine induced toxicity by its ability to inhibit lipid peroxidation through the free radical scavenging activity, which enhanced the antioxidant defense system.[26] As a possible molecular mechanism for the antioxidant action of THU, it has been suggested that the beta-diketone moiety of THU exhibits antioxidant activity by cleavage of the C-C bond at the active methylene carbon between two carbonyls in the beta-diketone moiety [23], [60].

Cd accumulates mainly in the liver, kidneys, heart and aorta which cause tissue damage in these organs [14], [61]-[62]. We found that the Cd content in these organs and the blood was decreased in Cd exposed mice with THU co-administration. The large reduction in Cd concentration in the blood after THU treatment suggests that THU, like curcumin, may be able to chelate Cd by forming a metal-ligand complex, thereby reducing the Cd load in the body [63]. A potential chelating effect of THU at the tested dose is supported by the improvement of hemodynamic and vascular responses to a great extent; in particular a low dose of THU improved those variables. Moreover, data from supplementary experiments revealed the chelating and antioxidant properties of THU in mice receiving Cd directly via intravenous injection. Apart from being a chelating and antioxidant agent, THU might also have the ability to interfere with the gastrointestinal absorption of Cd, thereby, causing a reduction in Cd content in the blood and tissues. However, the specific mechanism is not clear and deserves further investigation.

In conclusion, the results obtained in this study provide the first experimental evidence that THU protects against hypertension, increased arterial stiffness, vascular remodeling and oxidative stress during Cd intoxication. The data suggest that THU as a chelating and antioxidant agent plays an important role in lessening the adverse effects of exposure to Cd. Although the results of this study revealed the protective effect of THU during Cd exposure, the therapeutic effect of THU after Cd exposure is another important aspect and merits further detailed investigation. Collectively, these findings suggest a beneficial effect in using antioxidant THU as a dietary supplement following heavy metal exposure, or as a complimentary chelating agent to increase the efficacy of a known chelator in order to minimize the metal's toxicity.

## Supporting Information

S1 Figure **Effect of THU on mice acutely exposed to Cd by intravenous injection.** Cd treated group, mice received CdCl_2_ (100 µg/ml) in saline by intravenous injection via the tail vein, once daily for 3 days; the injection volume was 200 µl/mouse. Cd+THU treated group, mice were intravenously administrated with Cd once daily, and THU (372 µg/ml) in 0.5% DMSO, for 2 times a day (30 min after Cd injection and in the evening) for 3 days. Control group, mice were injected with saline and 0.5% DMSO, 2 times a day for 3 days. Results are expressed as mean ± SEM., n = 3/group. ^*^ *P*<0.05 compared with normal control group, #*P*<0.05 compared with Cd control group.
(TIF)
