# Correction: Tetrahydrocurcumin Protects Against Cadmium-Induced Hypertension, Raised arterial Stiffness and Vascular Remodeling in Mice

**DOI:** 10.1371/journal.pone.0118755

**Published:** 2015-03-05

**Authors:** The PLOS ONE Staff

There is an error in the legend for Figure 3 “Effect of THU on the relative wall thickness (A), circumferential extension ratio (B), E_p_ (C), and E_inc_ (D) of mice thoracic aortas in all experimental groups”. The complete, correct Figure 3 legend is:

**Figure 3. Results are expressed as mean ± SEM., n = 6–8/group.** In animals not treated with Cd, THU has no effect on aortic elasticity of normal control mice (data not shown). (A)*P<0.05 compared with normal control group at various pressures. ^†^ *P*<0.05 compared with Cd control group at various pressures. (B)*P<0.05 compared with normal control group at pressures ranging from 160–200 mmHg; ^‡^ *P*<0.05 compared with normal control group at pressures ranging from 180–200 mmHg); ^†^ *P*<0.05 compared with Cd control group at pressures ranging from 160–200 mmHg. (C)*P<0.001 compared with normal control group at pressures ranging from 100–200 mmHg; ^‡^ *P*<0.05 compared with normal control group at pressures ranging from 120–200 mmHg; ^†^ *P*<0.05 compared with Cd control group at pressures ranging from 120–200 mmHg. (D)*P<0.05 compared with all group (from 1.7);^‡^ *P*<0.05 compared with normal control (from 1.9); ^†^ *P*<0.05 compared with Cd control group (from 1.7).

In Fig. 6D, the second column (indicated as Cd) is missing an asterisk. Please see the corrected Fig. 6D here.

**Fig 6:**
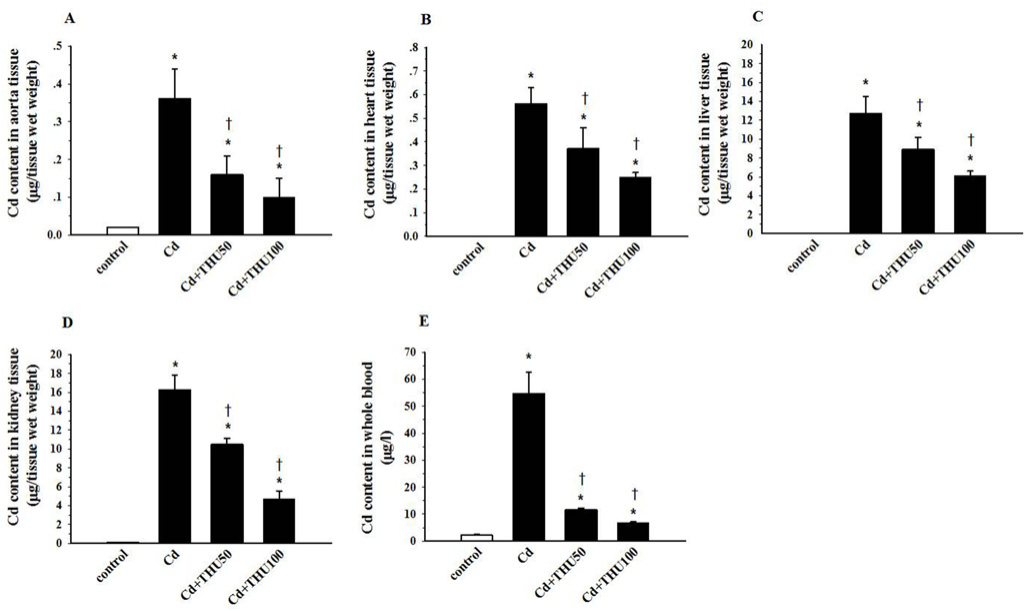
Effect of THU on Cd accumulation in the blood and tissues of mice exposed to Cd. Results are expressed as mean ± SEM., n = 6–8/group. *P<0.05 compared with normal control group,^†^ *P*<0.05 compared with Cd control group.
