# Supplementary figures and images for: Tetrahydrocurcumin Protects against Cadmium-Induced Hypertension, Raised Arterial Stiffness and Vascular Remodeling in Mice
Source: PLoS One. 2014 Dec 11;9(12):e114908. doi: 10.1371/journal.pone.0114908 (PMC4263715; doi:10.1371/journal.pone.0114908)

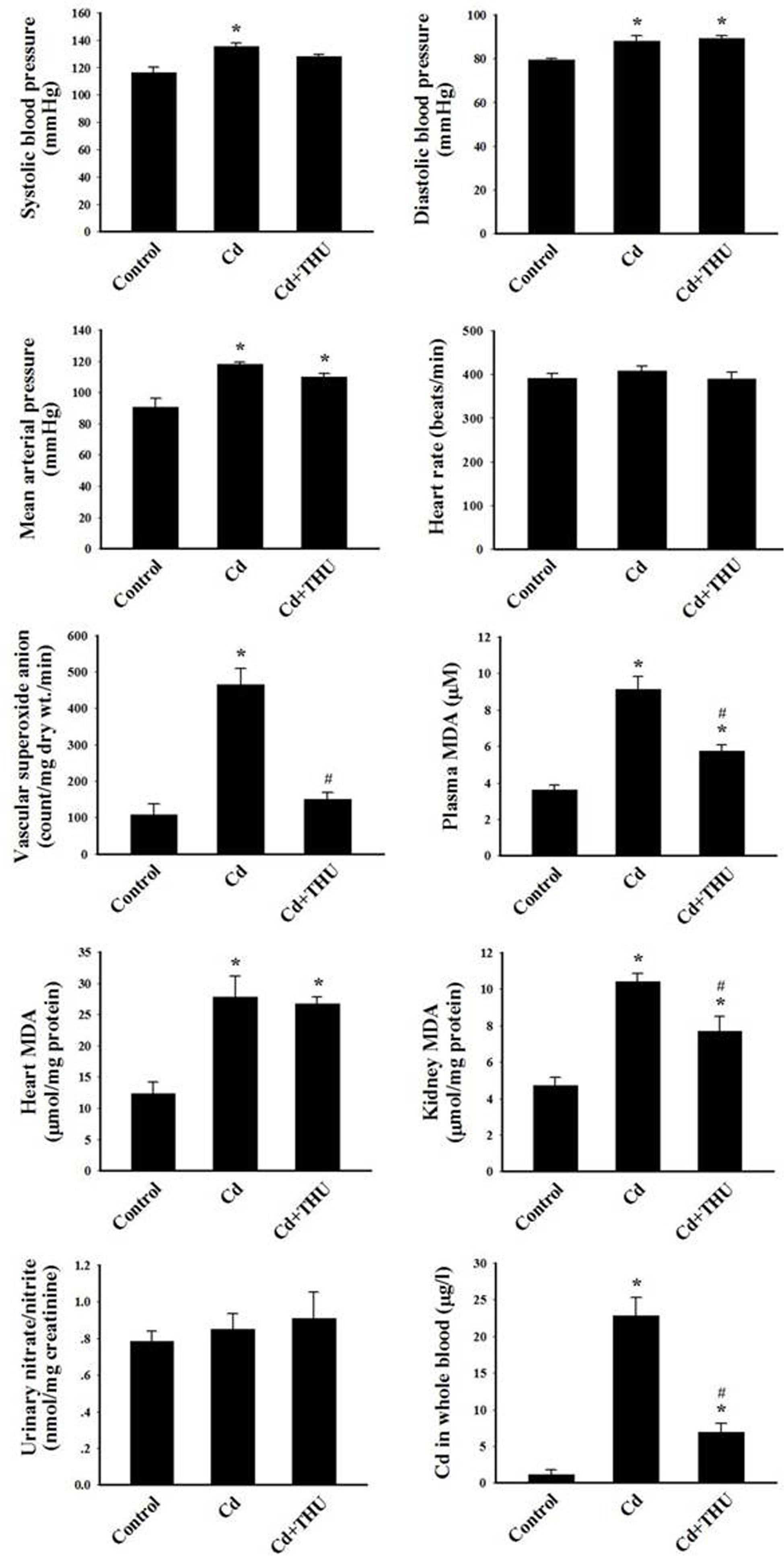

Supplement: S1 Figure — Effect of THU on mice acutely exposed to Cd by intravenous injection. Cd treated group, mice received CdCl2 (100 µg/ml) in saline by intravenous injection via the tail vein, once daily for 3 days; the injection volume was 200 µl/mouse. Cd+THU treated group, mice were intravenously administrated with Cd once daily, and THU (372 µg/ml) in 0.5% DMSO, for 2 times a day (30 min after Cd injection and in the evening) for 3 days. Control group, mice were injected with saline and 0.5% DMSO, 2 times a day for 3 days. Results are expressed as mean ± SEM., n = 3/group. * P<0.05 compared with normal control group, #P<0.05 compared with Cd control group. (TIF) [file pone.0114908.s001.tif]
